# Where’s the risk? Landscape epidemiology of gastrointestinal parasitism in Alberta beef cattle

**DOI:** 10.1186/s13071-015-1040-x

**Published:** 2015-08-25

**Authors:** Melissa A. Beck, Douglas D. Colwell, Cameron P. Goater, Stefan W. Kienzle

**Affiliations:** Department of Biological Sciences, University of Lethbridge, 4401 University Drive, Lethbridge, AB T1K 3 M4 Canada; Agriculture and Agri-Food Canada, Lethbridge Research Station, 5403 1st Ave South, Lethbirdge, AB T1J 4B1 Canada; Department of Geography, University of Lethbridge, 4401 University Drive, Lethbridge, AB T1K 3 M4 Canada

**Keywords:** Gastrointestinal nematodes, GIS, Bayesian, Multivariate hierarchical models, Cattle

## Abstract

**Background:**

Gastrointenstinal nematodes (GIN) present a serious challenge to the health and productivity of grazing stock around the globe. However, the epidemiology of GIN transmission remains poorly understood in northern climates. Combining use of serological diagnostics, GIS mapping technology, and geospatial statistics, we evaluated ecological covariates of spatial and temporal variability in GIN transmission among bovine calves pastured in Alberta, Canada.

**Methods:**

Sera were collected from 1000 beef calves across Alberta, Canada over three consecutive years (2008–2010) and analyzed for presence of anti-GIN antibodies using the SVANOVIR *Ostertagia osteragi*-Ab ELISA kit. Using a GIS and Bayesian multivariate spatial statistics, we evaluated the degree to which variation in specific environmental covariates (e.g. moisture, humidity, temperature) was associated with variation in spatial and temporal heterogeneity in exposure to GIN (*Nematodirus* and other trichostrongyles, primarily *Ostertagia* and *Cooperia*).

**Results:**

Variation in growing degree days above a base temperature of 5 °C, humidity, air temperature, and accumulated precipitation were found to be significant predictors of broad–scale spatial and temporal variation in serum antibody concentrations. Risk model projections identified that while transmission in cattle from southeastern and northwestern Alberta was relatively low in all years, rate of GIN transmission was generally higher in the central region of Alberta.

**Conclusions:**

The spatial variability in risk is attributed to higher average humidity, precipitation and moderate temperatures in the central region of Alberta in comparison with the hot, dry southeastern corner of the province and the cool, dry northwestern corner. Although more targeted sampling is needed to improve model accuracy, our projections represent an important step towards tying treatment recommendations to actual risk of infection.

## Background

The distribution, occurrence, and intensity of parasites varies enormously between samples of hosts from different sites, seasons, and years, in part due to interspecific sensitivity of infective stages to variable environmental conditions [1]. However, an incomplete understanding of the epidemiology of many direct and indirect-lifecycle parasites continues to limit the identification of high-risk locations and peak transmission periods. To address these key knowledge gaps, landscape epidemiologists seek to characterize variability in rates of parasite transmission in the context of changing climatic and landscape characteristics that arise naturally or through anthropogenic modification [2]. Advances in this area have often involved the use of modern Geographical Information Systems (GIS) tools, statistical modelling, and improved diagnostic techniques. Evaluation of spatial patterns for a number of vector-borne and other parasitic infections, including schistosomiasis (e.g. [3]) and malaria (e.g. [4]), have facilitated the prediction of transmission risk in unsurveyed areas, have directed large-scale intervention programs [5], and have helped predict future outbreaks relative to climate warming projections [6]. Despite advances in the use of these spatial tools (e.g. [7]), major gaps central to understanding spatial heterogeneity in gastro-intestinal nematode (GIN) transmission among domestic stock remain. The lack of accurate epidemiological data is especially acute in northern latitudes involving domestic stock as hosts [8].

GIN occur globally in grazing mammals, representing a significant threat to the sustainability of livestock production [9]. Infection is a common cause of reduced weight gain, intestinal dysfunction, dysentery, anorexia, and anaemia [10]. In Canada, livestock operations represent a significant component of the agrarian economy. As elsewhere, GIN control programs continue to rely on intensive anthelmintic use aimed at preventing the accumulation of parasite burdens over successive grazing seasons. This approach has been based on observed increases in host productivity following the application of anthelmintics [11, 12]. Macrocyclic lactone dosage for roundworm and ectoparasite control is associated with an estimated saving of $7.04 per head in calves and $4.2 per head in yearling cattle compared with control of ectoparasites alone [13, 14]. Despite these clear production and health benefits, the blanket treatment of animals can result in the overuse of anti-parasitics. This gives rise to the threat of anthelmintic resistance affecting the ability to control these parasites and is associated with high costs to producers [9, 15].

An improved understanding of the influence of climatic characteristics on GIN transmission can aid in the development and implementation of evidence-based parasite control programs aimed at reducing this economic burden and reducing the risk of anthelmintic resistance. Each species of GIN has critical temperature and moisture requirements for optimal development, beyond which development slows and the likelihood of larval survival declines [16]. Suboptimal environmental conditions, such as temperature and moisture extremes, that impact the distribution and survival of free-living larval stages (e.g. [17, 18]), likely resulting in variable transmission across the landscape and over time. At present, little information is available regarding the broad-scale environmental factors that influence the availability of GIN larvae on pasture in northern latitudes. The use of GIS for the development of broad-scale statistical models is therefore valuable for prediction of risk of GIN transmission and in providing an ecologically grounded baseline for management.

Here we focus on improving our understanding of heterogeneity in risk of GIN in domestic beef cattle at a province-wide scale. Our objectives were to: 1) define the temporal and spatial variability in GIN transmission across the province of Alberta, Canada; 2) use a GIS-based approach to evaluate the broad-scale environmental covariates of spatial and temporal heterogeneity in transmission; and 3) create a model to predict risk of infection. We combined standard indirect measures of parasite transmission (ELISA detection of anti-GIN antibody concentrations) with GIS technology to characterize variability in GIN exposure over three consecutive years in bovine calves. Bayesian inference was used to model variability in parasite exposure in relation to key environmental characteristics.

## Methods

### Study area

**Fig. 1:**
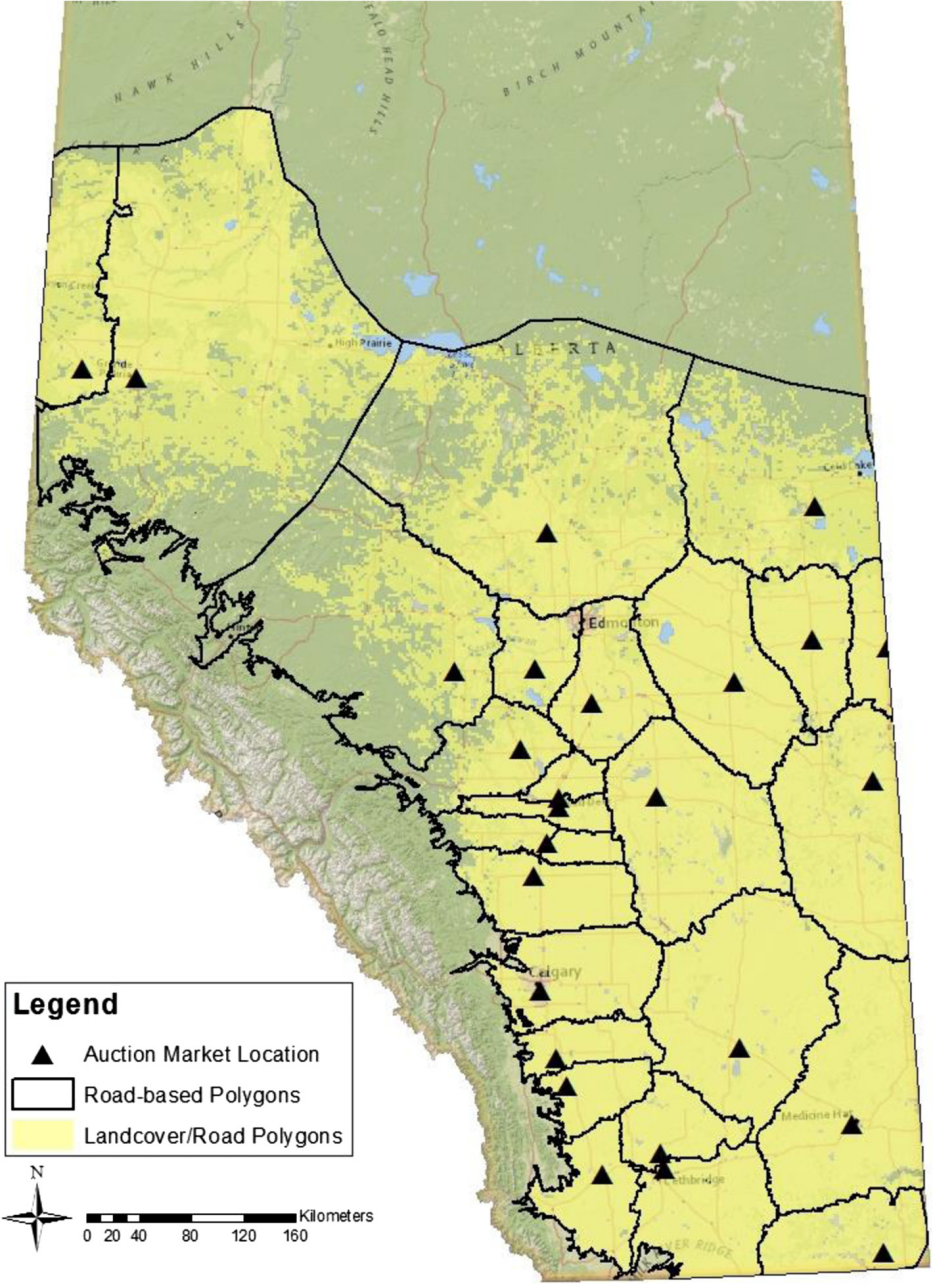
Sampling polygons for GIN survey in southern Alberta bovine calves. Southern Alberta was delineated into 26 service area polygons based on analyses of minimum driving distance to auction markets in accordance with the existing road network.

The province of Alberta extends from 49 to 60° latitude north, with an area of approximately 661,848 square km. The province has three major biogeographical divisions ranging from west to east which vary in elevation and associated climate: the mountains, the foothills, and the plains [19]. Our study area is focused on the 79, 000 square km plains region where grazing on native rangelands and Crown and community pastures is most extensive (Fig. 1) [19]. The plains region comprises the majority of the total area of the province, with elevation varying from 800 m along the eastern border of the province to approximately 1800 m along the foothills belt in the west [20]. The southeastern corner of this region has an average annual precipitation (1971–2000) of 331 mm (CV: 8.4 %), and an annual maximum temperature (1971–2000) of 21.7 °C (CV: 0.3 %) during peak grazing season (Jun to Oct) that is associated with a high rate of evapotranspiration, frequent hot dry winds, and prolonged periods of low precipitation. Further north, the annual precipitation increases to about 515 mm (CV: 7 %) in the centre of this zone, and then decreases to 475 mm (CV: 6.0 %) in the far northwest and 487 mm (CV: 13.0 %) in the northeast. Evapotranspiration likely decreases with a maximum annual temperature of 16.8 °C (CV: 0.2 %) during the grazing season in the northwest of this region and 18 °C (CV: 0.2 %) in the northeast. Average precipitation also increases markedly from east to west, with approximately 368 mm (CV: 7.7 %) of rain along the eastern boarder of the province to as much as 467 mm (CV: 8.5 %) on the edge of the foothills [21].

### Hosts

In fall (November through December) of 2008–2010, 1000 cross-bred (Angus cows X Hereford bulls) and purebred Angus calves were sampled from a total of 26 auction markets across Alberta. Auction markets were distributed throughout the study region and were opportunistically sampled. Calves were sampled by feedlot staff working in conjunction with Feedlot Health Management Services and researchers from Agriculture and Agri-food Canada. We targeted beef calves coming off their first year on pasture to minimize variation in egg counts due to host age and immunity. We also restricted the sampling window to a 6-week period each fall to minimize heterogeneity due to inherent seasonal variation. Calves were born in April-May of each year and were maintained on pasture with their dams until weaning in November through December. Calves are transitioned to a finishing diet upon entry into a feedlot or go into a feeding program to prepare them for grazing in their second year [22]. All cattle were sampled prior to anthelmintic treatment, with a 10 % random sample [22] of calves selected from each sampled “lot” (auction market). Animals were handled under the guidelines of the Canadian Council for Animal Care (Animal Care Committee protocol # 08233, 0925 and 1044).

### Faecal egg counts

The presence of eggs in samples of faeces was used to identify the GIN spp. present in each host. Faeces were collected by rectal palpation, stored in individual labelled bags, and frozen prior to analysis. A modified Wisconsin technique with a sugar solution [23] was used to process faecal samples. Parasite eggs were identified to genus (*e.g. Nematodirus* spp.) according to descriptions in Olsen [24]. Due to similar egg morphologies, all trichostrongyle genera were pooled and termed ‘trichostrongyles’.

### Serum antibody concentrations

Blood was collected by jugular venipuncture into vacutainer tubes with serum separators (BD-Canada Inc., ON) from each calf, analyzed using SVANOVIR® *Ostertagia ostertagi-* Ab ELISA kits (Boehringer Ingelheim SVANOVA, Uppsala, Sweden). The reference sera were diluted 1:140 [25]. Optical density values read at 405 nm were standardized as an optical density ratio (ODR) using negative and positive control sera samples included on each plate.

### Mapping and meteorological data

All GIS-based mapping analyses were completed in ArcGIS, version 10.1 (Source: ESRI). Spatial analysis required the following digital data sources: digital elevation model (DEM, source: Geobase), generalized land cover map (source: DB Geoservices Inc.), road network (source: ESRI), geo-referenced auction market locations (Fig. 1), and climate data (source: Alberta Agriculture and Rural Development: http://agriculture.alberta.ca/acis/alberta-weather-data-viewer.jsp). For visualization, the Alberta base map was obtained from free sourced data made available in joint by National Geographic, Esri, De Lorne, NAVTEQ, UNEP-WCMC, USGS, NASA, ESA, METI, NRCAN, GEBCO, NOAA, and IPC.

Precise coordinates for grazing pastures were not available. Thus, we calculated the likely service area for each individual lot. These service area polygons were created using the existing road network around each georeferenced lot location, making the assumption that producers select an auction market based upon minimum driving distance. We assumed that unknown sources of error, including lot preferences of producers, cancel each other out. Areas in the province where grazing is not common were excluded based on elevation (over 1250 m), land cover type (e.g. coniferous forests, lakes), and presence of urban development (Fig. 1).

Meteorological data were averaged from all geo-referenced climate stations falling within each polygon. The environmental variables considered in the study, especially those associated with temperature and moisture availability, were selected based upon their known role in determining nematode viability and infectivity [17, 26, 27]. We only used same-year environmental data, as overwinter larval survival and development of eggs is unlikely in this region [28]. It is therefore assumed that GIN exposure is related to the seeding of pasture in the spring by dams infected during the previous grazing season(s).

Environmental data were collected from May to October to represent the growing season prior to the collection of faecal and serum data at sacrifice [29]. This temporal period represents the development period of larvae shed when adult cattle are returned to pasture in May of each year, typically followed by peak GIN intensities in cattle and on grazing pasture during the summer months [30]. Data were obtained for the following periods: May–September, June–September, July–September, August–September, May–October, June–October, July–October, and August–October. These data included: (i) total accumulated precipitation (mm), (ii) average daily accumulated precipitation (mm); (iii) average, minimum, and maximum air temperature (°C), (iv) average, minimum, and maximum relative humidity (%), (v) total accumulated growing degree days (GDD) with a base 5 °C, and (vi) average daily growing degree days (GDD) with a base 5 °C. Relative humidity is a dimensionless ratio, expressed in percent, of the amount of atmospheric moisture present relative to the amount that would be present if the air were saturated. Since the latter amount is dependent on temperature, relative humidity is a function of both moisture content and temperature. Accumulated GDDs were calculated as the accumulation of days with an average daily temperature exceeding 5 °C for each of the stated temporal periods. Mean daily GDD is an average of the daily increase in GDD with a base temperature of 5 °C for each weather station.

### Statistical analyses

ODR data were normalized by log (n + 1) transformation. Due to cross antigenicity, *O. ostertagi*- Ab ELISA kits are indicative of exposure to a number of GIN genera [7]. Chi-squared statistics were used to compare prevalence (p) between samples, with 95 % confidence intervals (CI) calculated using the Wald method (p+/− z√(pq/n), where z = 1- alpha/2 of the standard normal distribution and q = 1-p) [31]. Mean ODR (± SEM) values for each polygon were compared using ANOVA with Tukey’s post hoc comparisons for each sampling year.

For hypothesis testing, environmental data were paired with mean ODR values from each polygon. Variables were standardized by subtracting the mean and dividing by two standard deviations [32]. This conversion accounts for differences in dimension and variance, improves the efficiency of the sampling algorithm, and has no effect on the resulting model.

Bayesian inference was used to construct hierarchical logistic regression models in OpenBUGS version 3.2.2 [33] to test each environmental variable separately, and in combination, for each of the eight temporal periods. The main advantage of the Bayesian approach is that parameter uncertainty is fully accounted for when performing prediction and inference, even when sample sizes are small. With a hierarchical Bayesian approach we obtain a full accounting of variability among individual polygons, years of sampling, and other environmental covariates, together with estimates of observation errors [34, 35]. Risk of transmission was modelled as a linear function on a log scale. A non-informative prior distribution (mean = 0, tau = 1.0 X 10^−4^) was assigned to the regression coefficients. Sampling year, assumed to follow a uniform normal distribution was included in all models as a random effect.

For all models, we discarded the first 60,000 iterations, with another subsequent 40,000 iterations used to estimate model parameters. This initial burn-in was required to ensure that the model chains converged and that the parameter space has been correctly explored [34]. Competing models were ranked by their deviance information criterion (DIC), which is a measure of model fit to the data. The best model is that with the lowest DIC value. To compare models, the difference between the DIC_*i*_ of each model and the DIC_*i*_ of the best fit model (minDIC_*i*_) was calculated for each model:

$$ \varDelta \mathrm{D}\mathrm{I}\mathrm{C}={\mathrm{DIC}}_i\hbox{-} {\mathrm{minDIC}}_i $$

Models within two ΔDIC units of the top performing model were considered to have strong support, within four to seven ΔDIC units to have considerably less support, and greater than ten, no support [35, 36].

Expected ODR values (i.e. estimated risk of transmission) were calculated for each individual polygon using all the models within two ΔDIC of the top-performing model. Values were then averaged to obtain a mean expected ODR value for each polygon for each of the three sampling years. Using this approach, we account for model uncertainty [33]. Average annual expected transmission risk was assigned values corresponding to low, moderate and high mean ODR values of <0.3, 0.3 to 0.5, and > 0.5, respectively. ‘High risk’ (ODR >0.5) was considered indicative of high rates of GIN exposure [37].

Temporal variability in environmental covariates was evaluated using ANOVA with Tukey’s post-hoc comparisons for polygons where risk of high GIN exposure varied between years. To assess the spatial accuracy of our model predictions, we compared model prjected ODR values with observed ODR values for each polygon using Chi-squared analyses. Parametric correlation coefficients were obtained comparing observed mean ODR values and model projected ODR using data pooled for all three years. Mean square error was then calculated to assess model accuracy.

### Model validation

To validate the GIN transmission risk model, we targeted two polygons for follow-up analyses in 2013. Based on our model projections, one polygon had consistently low risk of GIN transmission, whereas a second had moderate to high risk of economically significant parasite transmission. The Agriculture and Agri-Food Canada, Lethbridge Research Centre (LRC) field station located at One Four, Alberta (49.4° N, 110.7° W) was selected as the low risk site, while a ranch near Stettler, Alberta, was the moderate to high risk site. Blood was collected from each calf by jugular venipuncture and analyzed as outlined above for a total of 167 cross-bred calves from the LRC field station and 75 calves from the Stettler ranch.

For initial comparison of parasite transmission differences between the two ranches we calculated: (1) mean ODR (± SEM) for each ranch; (2) proportion of calves with bootstrapped 95 % CI that were parasite negative (ODR < 0.0); and (3) proportion of calves with bootstrapped 95 % CI with high intensity infections (ODR > 0.5) [36]. Mean values were compared using parametric t-tests, and differences in prevalence values were evaluated using Chi-squared.

Climate data for 2013 were obtained from the Alberta Agriculture and Rural Development. Using these environmental data, mean (± SEM) expected ODR values were calculated using the top performing models. We compared model projections based on data obtained from the closest climate station to each respective ranch using Euclidean straight-line distances. We then validated our model using parametric t-tests to compare observed and expected ODR values. Parametric t-tests are also used to compare environmental means used in calculating model projections of exposure risk between these two sites.

## Results

### Infection patterns

**Fig. 2:**
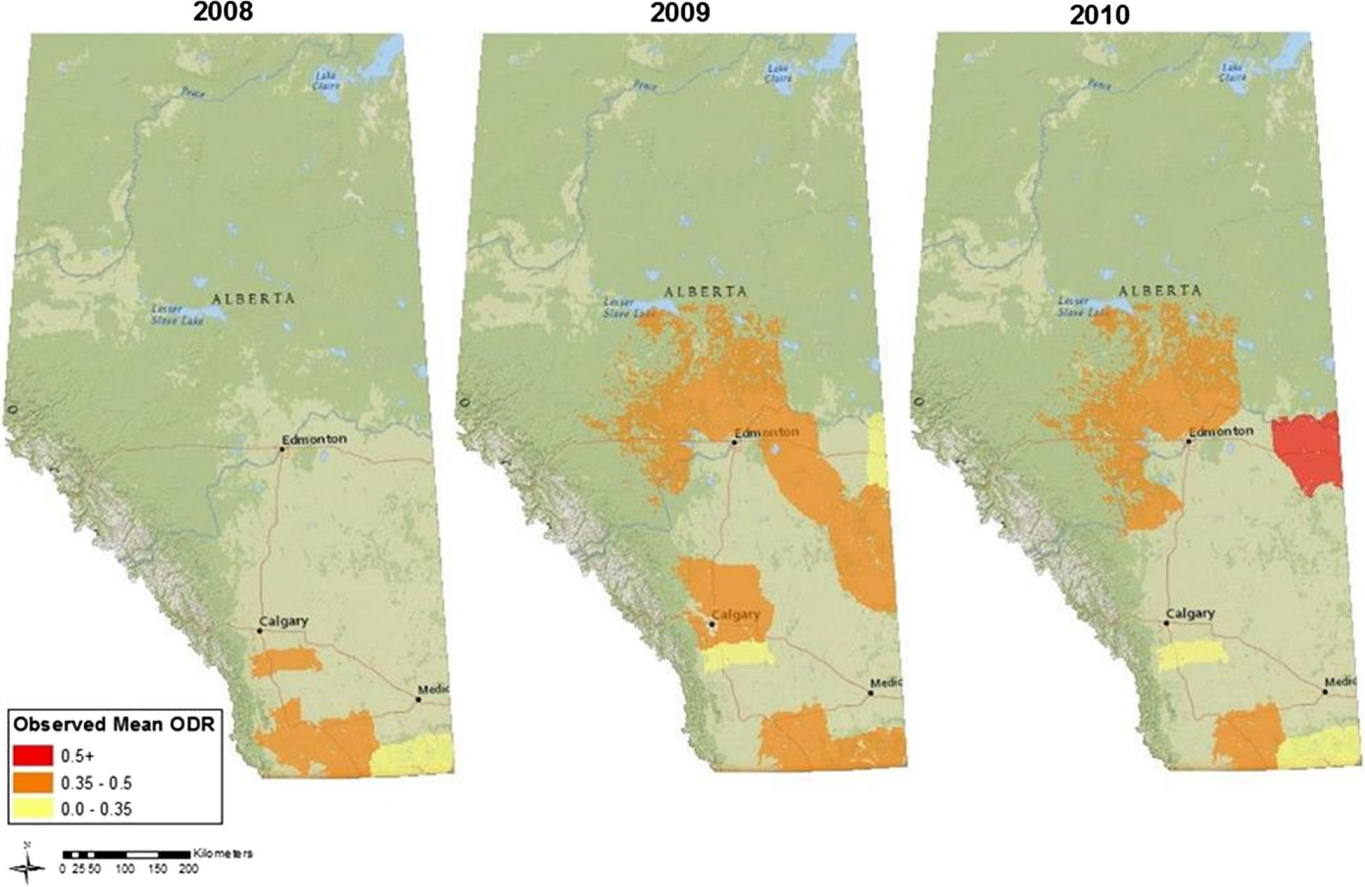
Observed mean antibody concentrations (ODR) in calves sampled at auction markets in Alberta from 2008 to 2010

97.2 % (95 % CI: 96.2-98.3 %) of the 1000 calves sampled from 2008-2010 were sera-positive for GIN. Although the overall proportion of sera-positive animals remained consistent between years, and estimated prevalence did not significantly vary among individual polygons in 2008 (*χ*^2^ = 3.1, *p* = 0.86) and 2010 (*χ*^2^ = 5.7, *p* = 0.86), spatial variation in mean ODR was significant in all three years (Fig. 2; 2008: F_3,281_ = 24.4, *p* < 0.001; 2009: F_9,321_ = 2.4, *p* < 0.05; 2010: F_7,237_ = 5.7, *p* < 0.001). The proportion of infected hosts varied significantly among polygons in 2009 (*χ*^2^ = 35.2, *p* < 0.01), however, this variation can be accounted for by an increase in the number of sera-negative animals (n = 20) from the LRC ranch. Data from five polygons that were sampled in all three years also showed significant annual variation in transmission. These include data from the following five auction markets presented in Fig. 1: High River, Innisfail, Lethbridge, Lloydminster, and OneFour, Alberta. Colwell *et al*. [25] reported significant variation in ODR between years for cattle sampled from the LRC ranch at OneFour, Alberta (N 50.5° W −113.4°), with values significantly higher in 2009 in comparison with 2008 and 2010. Despite significant variation, risk of infection was consistently low (ODR < 0.35) in this southeastern corner of the study area (Fig. 2). Annual variation in ODR for cattle from the High River area paralleled results observed at the LRC ranch, while in 2009 ODR values were significantly lower in the Lloydminster polygon in comparison with other sampling years. In contrast, annual differences in mean ODR were not detected for a polygon along the southern border of the province (Lethbridge), nor were annual differences detected in the center of our survey area (Innisfail).

Of the > 1000 models run, three additional models were within two ΔDIC of the top-performing model. Various combinations of the following variables were found to be significant predictors of transmission risk in these models (Table 1): minimum air temperature, average daily growing degree days with a base of 5 °C, accumulated precipitation, daily average accumulated precipitation, and minimum humidity. These top models all relied on environmental data collected from July to October of each respective grazing year. Models calculated using environmental data for the other seven temporal periods did not perform equally as well.

**Table 1:** Summary of top performing multivariate hierarchical models for risk of GIN transmission. Models are ranked based on the Deviance Information Criterion (DIC). Year is included in all models as a random fixed effect

Rank	Model Parameters	DIC	ΔDIC
1	Daily average GDD (base 5 °C), Minimum temperature (°C)	54.71	0.00
2	Daily average GDD (base 5 °C), Minimum temperature (°C), Daily accumulated precipitation (mm/day)	56.02	1.31
3	Daily average GDD (base 5 °C), Minimum temperature (°C), Total accumulated precipitation (mm)	56.11	1.40
4	Daily average GDD (base 5 °C), Minimum temperature (°C), Minimum humidity (%)	56.27	1.56
Null Model	-	106.20	51.49

**Fig. 3:**
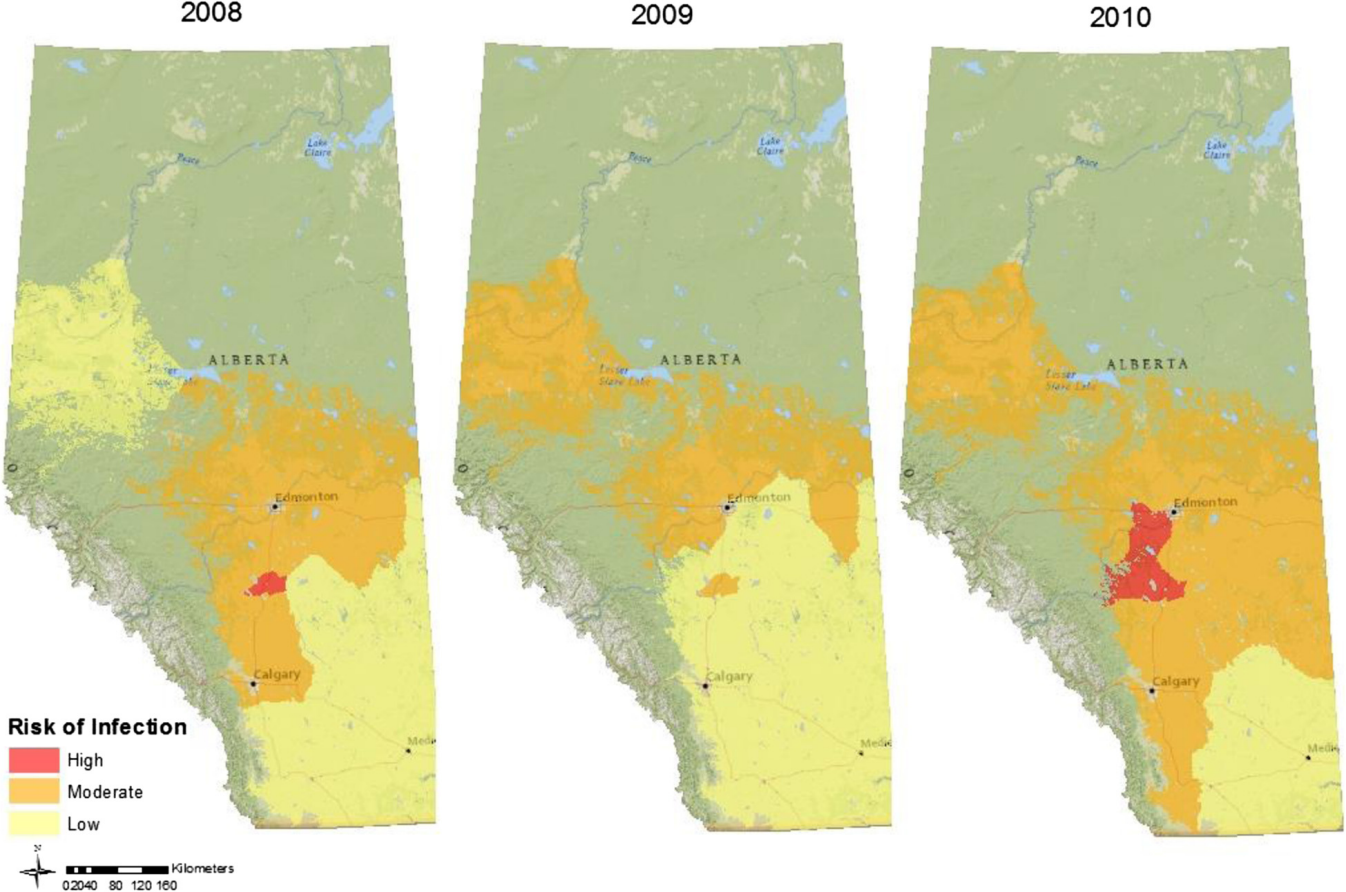
Model predicted spatial and temporal variation in risk of GIN transmission in Alberta bovine calves (2008–2010). Distribution of expected risk of nematode transmission calculated for each year using Bayesian inference to construct hierarchical binary response logistic regression models for ODR in cattle sampled at auction markets in southern Alberta from 2008 to 2010. Low, Moderate and High risk are differentiated according to mean regional optical density ratio values of <0.3, 0.3-0.5, and >0.5 respectively for cattle serum

Expected ODR was calculated for each of the 4 models (Table 2), averaged to account for model uncertainty [35] and projected across the study area for all three years (Fig. 3). Model projected risk was consistently low (ODR <0.35) in the far southeast. The total area where risk of economically significant infection was high increased in 2010 in comparison with 2008. This change may be attributed to a general increase in accumulated precipitation, fewer total GDDs, lower average maximum temperature, and higher minimum average temperature (Table 3). In comparison, total number of GDD was notably higher in these same polygons in 2009, with maximum temperature ranges similar to that of 2008.

**Table 2:** Regression model for tests of associations between means of regional environmental data and serum antibody concentrations (ODR)

Model	Variable	Parameter Mean	SD	B	SE B
1	Intercept	-			
	Daily Average GDD (base 5 °C)	8.06	1.08	−0.40	<0.01
	Minimum Temperature (°C)	5.29	1.01	0.20	<0.01
2	Intercept	-	-	−0.64	<0.01
	Daily Average GDD (base 5 °C)	8.06	1.08	−0.45	<0.01
	Accumulated Precipitation (mm)	153.31	40.49	−0.05	<0.01
	Minimum Temperature (°C)	5.29	1.01	0.22	<0.01
3	Intercept	-	-	−0.64	<0.01
	Daily Average GDD (base 5 °C)	8.06	1.08	−0.05	<0.01
	Daily Accumulated Precipitation (mm)	1.25	0.33	−0.45	<0.01
	Minimum Temperature (°C)	5.29	1.01	0.22	<0.01
4	Intercept	-	-	−0.64	<0.01
	Daily Average GDD (base 5oC)	8.06	1.08	−0.45	<0.01
	Humidity Minimum	38.34	7.15	−0.05	<0.01
	Minimum Temperature (°C)	5.29	1.01	0.22	<0.01

*SD* standard deviation, *B* parameter coefficient, *SE B* standard error of the coefficient

**Table 3:** Spatio-temporal patterns of environmental covariates of variability in GIN transmission risk on Alberta pasture between 2008 and 2010

Parameter	Polygon	Mean ± SEM			ANOVA
		2008	2009	2010	
GDD Total (base 5 °C)	8	867.5 ± 10.0	979.5 ± 46.5	865.1 ± 35.0	F_735,2_ = 3.8
	10	814.5 ± 31.7	852.4 ± 28.2	803.2 ± 12.4	F_996,2_ = 1.0
	12	795.8	799.6	726.2	-
	13	749.2	769.6	663.3	-
	14	865.7 ± 21.0	902.9 ± 28.1	804.9 ± 15.6	F_729,2_ = 4.9
	15	937.6 ± 18.6	950.4 ± 18.0	855.0 ± 15.4	F_4788,2_ = 8.9^***^
	18	881.8 ± 25.0	892.2 ± 22.7	812.1 ± 23.1	F_673,2_ = 3.4
	19	875.4 ± 17.5	893.6 ± 13.2	803.2 ± 12.4	F_1104,2_ = 10.8^**^
Acc. Precip. (mm)	8	98.6 ± 8.0	204.4 ± 32.7	158.8 ± 28.3	F_735,2_ = 4.4
	10	181.4 ± 10.3	137.2 ± 12.0	178.2 ± 13.9	F_996,2_ = 5.3^*^
	12	175.8	190.3	174.3	-
	13	181.3	206.6	285.2	-
	14	144.6 ± 16.5	190.3 ± 7.4	271.7 ± 16.5	F_729,2_ = 20.7^*^
	15	127.46 ± 7.5	183.4 ± 7.1	217.3 ± 8.5	F_4788,2_ = 34.4^***^
	18	113.3	124.1 ± 9.8	208.0 ± 21.6	-
	19	114.9 ± 12.3	120.0 ± 6.8	210.9 ± 21.8	F_1104,2_ = 13.0^**^
Maximum Temp. (°C)	8	20.8 ± 0.4	19.6 ± 0.6	19.0 ± 0.4	F_735,2_ = 3.5^*^
	10	19.3 ± 0.32	18.5 ± 0.5	17.8 ± 0.3	F_996,2_ = 3.5^*^
	12	18.7 ± 0.6	17.8 ± 0.8	17.4 ± 0.6	F_366,2_ = 1.0
	13	18.7 ± 0.6	17.7 ± 0.8	16.7 ± 0.4	F_489,2_ = 3.6^*^
	14	18.9 ± 0.4	17.8 ± 0.6	17.1 ± 0.4	F_729,2_ = 3.4^*^
	15	19.8 ± 0.2	18.9 ± 0.2	18.0 ± 0.2	F_4788,2_ = 22.9^***^
	18	19.4 ± 0.4	18.5 ± 0.4	17.5 ± 0.3	F_673,2_ = 5.4^**^
	19	19.2 ± 0.3	18.5 ± 0.4	17.5 ± 0.3	F_1104,2_ = 6.6^***^
Minimum Temp. (°C)	8	4.1 ± 0.4	4.6 ± 0.4	4.5 ± 0.3	F_735,2_ = 0.7
	10	3.2 ± 0.3	3.0 ± 0.3	3.3 ± 0.3	F_996,2_ = 0.3
	12	3.3 ± 0.5	2.8 ± 0.5	3.4 ± 0.5	F_366,2_ = 0.4
	13	2.3 ± 0.5	2.9 ± 0.5	3.0 ± 0.3	F_489,2_ = 0.8
	14	4.6 ± 0.4	4.8 ± 0.4	5.2 ± 0.3	F_729,2_ = 0.7
	15	4.5 ± 0.1	4.8 ± 0.2	5.2 ± 0.1	F_4788,2_ = 6.5^**^
	18	4.0 ± 0.4	4.7 ± 0.3	5.4 ± 0.2	F_673,2_ = 4.6^**^
	19	4.1 ± 0.3	3.8 ± 0.3	4.5 ± 0.2	F_1104,2_ = 2.1

*p < 0.05; **p < 0.01; ***p < 0.001

When data from all three years were pooled, a significant correlation was detected between mean observed ODR values and model projected ODR values (R = 0.46, *df* = 26, *p* < 0.05), with 18 % of the variance in ODR explained and a root mean square error of 0.082. Model predictions were more consistently accurate for polygons with intermediate risk on average, in comparison with polygons with a more extreme low or high mean ODR. The polygons with ‘extreme’ low or high ODR relative to the rest of the study area were characterized by higher variance in ODR values. Our top four models all included daily average GDD with a base temperature of 5 °C and minimum air temperature.

### Model validation

Mean ODR values differed significantly (*t*_240_ = −3.84, *p* < 0.001, *r* = 0.67) between the LRC ranch and the Stettler ranch (Table 4). The proportion of animals with ODR > 0.50 also significantly differed between sites (*χ*^2^ = 11.79, *p* < 0.001; Table 4) with higher ODR values on the Stettler ranch, indicative of an increased number of animals harboring high parasite counts.

**Table 4:** Comparison of observed and model-based projections of ODR in 2013. Projected values are based on data from the closest meteorological station in straight-line distance for ranches near One Four, Alberta and Stettler, Alberta

Site	N	Observed			Model Projected
		ODR (mean ± SEM)	Proportion of calves with ODR <0.0 (95 % CI)	Proportion of calves with ODR >0.5 (95 % CI)	ODR (mean ± SEM)
LRC	167	0.36 ± 0.02	0.05 (0.02-0.08)	0.25 (0.18-0.31)	0.16 ± 0.01
Stettler	75	0.51 ± 0.04	0.03 (0.01-0.05)	0.43 (0.35-0.50)	0.25 ± 0.01

Despite higher average daily precipitation on the LRC ranch in 2013 (Table 5) mean ODR was significantly higher in Stettler (Table 4; *t*_6_ = −7.6, *p* < 0.001). These data are consistent with our models with fewer GDDs, and higher minimum humidity associated with higher parasite intensities (Table 5). Mean expected ODR values did not significantly differ from observed mean ODR for the LRC ranch (*t*_169_ = 1.647, *p* = 0.101) and for the Stettler ranch (*t*_77_ = −1.536, *p* = 0.13). However, model projections did underestimated mean ODR at both locations (Table 4).

**Table 5:** Comparison of environmental data (July to October 2013) collected for model validation polygons. Environmental data are collected from the closest meteorological station by Euclidean straight-line distance for ranches near One Four, Alberta and Stettler, Alberta. Values are mean ± SEM

Environmental Parameters	LRC	Stettler	T-stat
Total Acc. Precip. (mm)	251.7	163.3	*-*
Daily Acc. Precip. (mm)	2.0 ± 0.6	1.3 ± 0.4	t_244_ = 9.5
GDD Total (base 5 °C)	1231.9	1012.9	*-*
GDD Daily Average (base 5C)	10.0 ± 0.6	8.2 ± 0.5	t_244_ = 2.3^*^
Air Temperature Minimum (°C)	7.8 ± 0.6	5.9 ± 0.5	t_244_ = 2.3^*^
Humidity Minimum (%)	36.0 ± 1.3	41.3 ± 1.4	t_244_ = −2.7^**^

*p < 0.05; **p < 0.01; ***p < 0.001

## Discussion

Our results show that almost all cattle in Alberta are exposed to at least one species of GIN in their first year. These data are consistent with previous empirical studies and survey reports of calves sampled from pastures in other north-temperate locations [16, 25]. Despite this ubiquitous presence, the relative risk of GIN exposure, as measured by antibody concentrations in host sera, varied significantly between polygons and between years.

A suite of environmental variables, likely acting in concert, explained a significant proportion of the overall variation in risk of exposure to GIN. Results from empirical laboratory studies and experimental studies involving tracer animals have shown that a large number of factors influence larval transmission rates from pasture into cattle. Thus, factors such as soil moisture, soil humidity, and air temperature may act at local scales in a species-specific and context-dependent manner [8, 16, 26]. Local-scale variation of this sort explains the tremendous variation that is typically observed between herds, even in cases where herds are adjacent on a landscape. These local factors likely contributed to the approximately 80 % unexplained variation in ODR values observed in this study. Yet despite this high level of background variation, the results of our study show that broad-scale variation in environmental factors that operate at the scale of 10’s or 100’s of kilometers explain a significant proportion of the overall variation in GIN infections in young cattle across Alberta.

The significance of the July to October temporal period in our top performing models is consistent with an increase in the availability of infective L3 on pasture throughout the grazing season [16]. Distinct seasonality has also been detected in a number of pasture-based studies with an increase in egg shedding to a peak in late August to early October [26]. This coincides with the peak in GIN spp. (e.g. *Ostertagia*, *Cooperia*, *Nematodirus*, and *Trichostrongylus* spp.) intensities detected in grazing tracer calves and dairy cattle in the fall and winter in northern temperate climates [29, 38]. Given these patterns, it follows that variability in environmental conditions during the July to October period would significantly impact the availability of L3 larvae on pasture.

Relative to spatial heterogeneity in nematode transmission, the significance of the number of GDDs may represent a required minimum number of days above a threshold temperature for larval development. This minimum requirement is also consistent with the significance of minimum air temperature, with risk generally increasing with higher average low temperatures. In the case of *O. ostertagi*, optimal temperatures for development range between 20 and 25 °C [26]. Rates of development slow, or may cease completely, as temperatures declines below this optima. Thus, a minimum number of GDDs may be needed for development, with the number of infective larvae available on pasture increasing in a given year with the number of cumulative GDDs. This pattern has been demonstrated for the development of *F. hepatica* on pasture [39]. However, at the other extreme, rates of development decrease when temperatures exceed the maxima for larval survival [26]. In a national survey of Canadian dairy cattle, Vanderstichel et al. [29] documented higher exposure to GIN on farms in areas with lower average land surface temperatures. The decreased risk of transmission with higher total GDDs may represent an increased number of consecutive days reaching beyond maximum threshold temperatures acting to limit parasite survival and development [17, 26, 27].

The significance of accumulated precipitation is also consistent with the transmission biology of infective 3rd stage larvae (L3) on pasture. While L3’s can survive for long periods within desiccated faeces, they cannot migrate vertically onto surrounding herbage in the absence of sufficient rainfall [18]. For *Haemonchus contortus*, an average of 2 mm daily rainfall failed to release substantial numbers of larvae [18]. In contrast, heavy rain has been found to yield high numbers of L3, with a daily average minimum of between 2 and 4 mm needed for larval migration on vegetation. Spatial variability in relative humidity can similarly affect the rate of desiccation of GIN eggs and free-living larval stages on pasture. These patterns are consistent with accounts of a cessation in larval development in the absence of sufficient moisture, regardless of the prevailing temperature [26]. Combined with our data, these results show that relative humidity and the amount and temporal distribution of rainfall are important drivers for GIN transmission, with the number and survival of free-living L3 on pasture influenced by regional precipitation patterns. Similar results were found in the assessment of environmental covariates of GIN in dairy cattle across Canada [29].

**Fig. 4:**
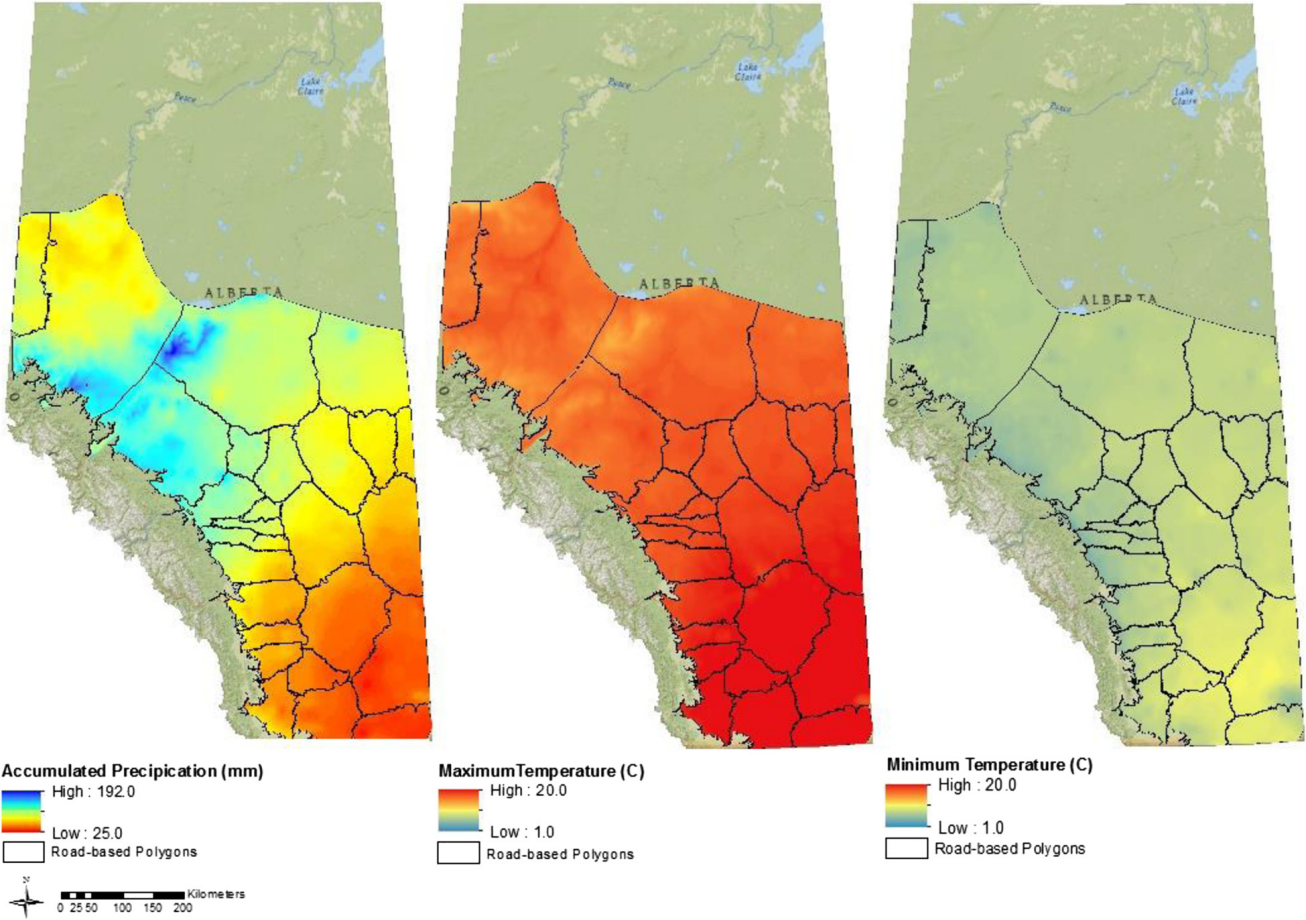
Alberta 30 year climate maps (1970–2000) for mean (i) accumulated precipitation (mm), (ii) maximum temperature (°C), and (iii) minimum temperature (°C) for July to October. Surface values were calculated as an average of monthly data created by Daly et al. [21]

Trade-offs between relative moisture conditions, temperature, and number of GDDs can help explain the spatial and temporal trends described in our risk maps. Low risk of L3 transmission was consistent in the southeastern and northwestern corners of the province. Average annual precipitation is generally higher in the northwest and lowest in the southeast (Fig. 4). The reverse is apparent for 30-year average annual temperatures [21] and number of GDDs for these regions. Data from the southeast are consistent with data from field and laboratory studies documenting that extremely arid conditions generally limit the development, survival and transmission of GIN. For example, the development of *O. ostertagi* L3 dropped from 30 to 5 % following an increase in temperature from 25 °C (optimal environmental conditions) to 32 °C [26]. In contrast, despite higher average annual precipitation in the northwest in comparison with the southeast, similarities in risk of transmission may be related to typically lower temperatures with fewer cumulative GDDs above a base temperature of 5 °C delaying larval development and limiting the number of infective larvae available on pasture [26, 39]. Stromberg [26] reports that *O. ostertagi* development rate is slowed at lower temperatures, taking up to 42 days at 5 °C. Other studies suggest that development may not occur at or below 5 °C [26]. We can therefore assume that just as the excessively dry and warm south and east parts of the province are not conducive to high rates of transmission, neither are the wetter, colder regions that are characteristic of the north and west. In comparison with these two relative extremes, intermediate temperatures and moisture availability characterize the transmission hotspot identified in the centre region of our study area.

Temporal variability in the area of high risk for economically significant transmission (Fig. 3) can also be attributed to differences between regions in accumulated precipitation, cumulative GDDs, and average temperatures. The increased total area in 2010 in comparison with 2008 is consistent with reports that increased precipitation, along with fewer consecutive days above maximum threshold temperatures for parasite survival and development, promote increased parasite transmission [17, 26, 27]. Similarly, the absence of high risk projections for 2009, along with overall expansion of the total area where risk was low along the eastern border of the province, can be explained by lower average precipitation and the increased GDDs that characterized that year’s unusually hot and dry summer.

Validation of our multivariate model demonstrated that projections at the regional level remained relevant in projecting ranch-specific risk. However, model projections did under estimate mean ODR values. This, as well as a large proportion of variation unaccounted for by our model, may be related to incidence of discrete rain events providing opportunity for drastic rise in availability in infective L3 [18]. These events were also presented as the most parsimonious explanation for high rates of L3 transmission in 2009 in an analysis of annual variation in serum antibody concentrations at the LRC ranch despite lower total accumulated precipitation [25].

The data for this study largely stemmed from regional-based parasite data. Information on the precise origin of host individuals, herd density and management strategies were not available. This limited the resolution and accuracy of our analyses. Additionally, our interpretations of risk were based on the assumption that detected antibodies (ODR) were indicative of exposure to a number of GI genera including *O. ostertagi* and *Trichostrongylus* spp. (see also 25). This assumption is supported by data from tracer calves in Alberta [40, 41]. Future research may benefit from the use of alternative diagnostic techniques that allow for the differentiation and quantification of specific parasite spp. present in each host [42]. Comparisons of more local-scale variation in risk of transmission using higher resolution spatial data may provide further insight into the epidemiology of parasite transmission. These data are important given that, despite life cycle similarities, species-specific sensitivity to environmental factors (e.g. tolerance of prolonged dry periods) is common (e.g. [18]).

## Conclusions

Despite the need to increase the overall robustness of model predictions, our model provides a baseline for evidence-based anthelmintic intervention. The data presented here demonstrate that in years with wetter- and warmer-than-average spring and summer conditions, we can expect higher rates of nematode transmission into yearlings in the fall, especially at sites in the center of our study area where transmission conditions appear to be optimal. Following further verification that links serum antibody concentrations to actual nematode burdens, these results can be used to guide future studies of GIN transmission biology and to maximize treatment efficiency [5, 43]. The next step is to attain more accurate data on: 1) parasite species-specific variation in intensity; 2) animal origin, history, and pasture characteristics; and 3) species-specific climate thresholds for GIN transmission. Of significance here will also be the determination of whether GIN can overwinter on pasture in this region. Such information will provide a platform for explaining species-specific distributional patterns and allow for optimization of anthelmintic applications. Combined with projected changes in climate, increased pressure on the landscape to support a growing global population, and rising incidence of anthelmintic resistance, the ability to reliably define variability in risk of parasite transmission will be increasingly important [43].

## Abbreviations

CV: Coefficient of variation
DIC: Deviance Information Criterion
ELISA: Enzyme-linked immunosorbent assay
ESA: European Space Agency
GEBCO: General Bathymetric Chart of the Oceans
GDD: Growing degree days
GIN: Gastrointestinal nematode
GIS: Geographic Information Systems
IPC: Integrated Food Security Phase Classification
LRC: Lethbridge Research Centre
METI: Maritime Environmental Training Institute
NASA: National Aeronautics and Space Administration
NOAA: National Oceanic and Atmospheric Administration
NRCAN: Natural Resources Canada
ODR: Optical density ratio
SEM: Standard error of the mean
UNEP-WCMC: United National Environmental Program – World Conservation Monitoring Centre
USGS: United States Geographical Survey
